# Educational Case: Hereditary breast and ovarian cancers

**DOI:** 10.1016/j.acpath.2023.100091

**Published:** 2023-09-07

**Authors:** Jerome P. Givi, Hannah W. Hazard-Jenkins, Melina Flanagan

**Affiliations:** a Department of Pathology, Anatomy, and Laboratory Medicine, West Virginia University, Morgantown, WV, USA; b West Virginia University Cancer Institute, Morgantown, WV, USA

**Keywords:** BRCA, Breast, Breast cancer susceptibility genes, Molecular basis of breast neoplasms, Organ system pathology, Pathology competencies

The following fictional case is intended as a learning tool within the Pathology Competencies for Medical Education (PCME), a set of national standards for teaching pathology. These are divided into three basic competencies: Disease Mechanisms and Processes, Organ System Pathology, and Diagnostic Medicine and Therapeutic Pathology. For additional information, and a full list of learning objectives for all three competencies, see https://www.sciencedirect.com/journal/academic-pathology/about/pathology-competencies-for-medical-education-pcme.^1^

## Primary objective

Objective BR2.4: Breast Cancer Susceptibility Genes. For the most common breast cancer susceptibility genes, describe the normal function of the gene product, incidence of gene mutation, reasons for its association with cancer, percentage of hereditary breast cancer, and risk of breast cancer by age 70.

Competency 2: Organ System Pathology; Topic BR: Breast; Learning Goal 2: Molecular Basis of Breast Neoplasms.

## Patient presentation

A 24-year-old woman presents to the clinic because her mother had been identified as a heterozygous carrier of a pathogenic *BRCA1* nonsense mutation, and she is interested in knowing her own cancer risk. The patient's mother had been diagnosed with invasive ductal carcinoma at the age of 48 years. She also reports a history of ovarian carcinoma in her aunt (mother's sister) at age 53, who is now 58 and whose disease is in remission, and invasive ductal carcinoma of the breast in her maternal grandmother, which was diagnosed at age 50. Her maternal grandmother passed away at age 52 because of the illness. The patient is nulliparous and has one 28-year-old brother with no medical problems. The remaining family history is negative.

The patient does not smoke and drinks 1–2 glasses of wine on the weekends. She is 162 cm tall and weighs 58 kg (BMI = 22.1). She jogs 4 miles 3–4 times per week, is sexually active with her partner of five years, and uses condoms for contraception.

## Diagnostic findings, Part 1

The patient appears anxious. Clinical breast examination reveals no masses or skin abnormalities. Nipples are rounded, everted, equal in size, and have no discharge. No palpable nodes are appreciated in the axilla. Clinical pelvic exam shows external genitalia without erythema, exudate, or discharge. No lesions are seen on the cervix, and the uterus is normal size with no masses. The adnexa are without masses or tenderness.

## Questions/discussion points, Part 1

### What are the molecular functions of the *BRCA1* and *BRCA2* genes, respectively?

*BRCA1* (breast cancer susceptibility gene 1) and *BRCA2* (breast cancer susceptibility gene 2) are tumor suppressor genes that code for proteins with roles in the repair of double-stranded breaks in DNA via homologous recombination. BRCA1 protein is also involved in the transcriptional regulation of several genes activated in response to DNA damage and plays a role in damage-responsive cell cycle checkpoints, as functional BRCA1 is required for effective S-phase and G2/M-phase checkpoints.^2^ Additional functions of the BRCA2 protein are less clear. Deleterious mutations in either of these genes can lead to aberrant cell replication and eventual cancer.

### What are the cancer risks associated with *BRCA1* and *BRCA2*, respectively?

Pathogenic germline variants of either *BRCA1* or *BRCA2* increase the risk of breast and tubo-ovarian cancers significantly. Females with *BRCA1* and *BRCA2* mutations have a 40–65% risk of developing breast cancer by age 70, compared to the 12% risk in the general female population.^3^ The risk of tubo-ovarian cancer is increased from 1 to 2% in the general population to 44% in *BRCA1* carriers and 17% in *BRCA2* carriers.^4^ Interestingly, while these genes have traditionally been considered to convey an increased risk for ovarian cancer, data now indicates that many of the related cancers are of fallopian tube origin.^5^ Overall, pathogenic germline variants of either *BRCA1* or *BRCA2* are estimated to account for 7% of all breast cancers and 14% of all ovarian cancer^6^^,^^7^ Additionally, *BRCA* carriers are at an increased risk of primary peritoneal carcinoma, a rare, aggressive malignancy that cannot be diagnosed with current screening tests,^8^ and risk of endometrial cancer may also be elevated. *BRCA* mutations, particularly *BRCA2*, increase the lifetime risk of breast cancer in males significantly, from approximately .01%–1% and 7% in *BRCA1* and *BRCA2* mutation carriers, respectively. In fact, the American Society of Clinical Oncology recommends all males diagnosed with breast cancer be tested for *BRCA* mutations.^9^ Pathogenic variants in either of the genes also increase the risk of prostate cancer in males. Mutations in either *BRCA* gene also increase the risk of pancreatic cancer in both males and females.^4^

### Germline mutations in what other genes are associated with breast cancer?

Although the *BRCA* genes are the most common and prominent genes associated with breast cancer, mutations in several other genes have also been linked to malignancy. These include genes associated with syndromic manifestations, including *TP53*, *PTEN*, *CDH1*, *STK11*, and mismatch repair genes, as well as non-syndromic genes such as *PALB2*, *CHEK2*, and *ATM*^10^^,^^11^ (see Table 1)**.** Those with mutations in these genes are at risk of certain types of cancers, and unique screening regimens must be followed in these patients.

**Table 1:** Genes related to breast cancer. Deleterious mutations in these genes cause increased risk of breast cancer, as well as other types of cancers.^10^^,^^11^

Gene	Encoded protein	Normal function	Syndrome	Associated tumors/conditions
*BRCA1*	BRCA1	Tumor suppressor	Hereditary breast and ovarian cancer syndrome	Breast, tubo-ovarian, endometrial, peritoneal, prostate, pancreatic, & male breast cancer.
*BRCA2*	BRCA2	Tumor suppressor	Hereditary breast and ovarian cancer syndrome	Breast, tubo-ovarian, endometrial, peritoneal, prostate, pancreatic, & male breast cancer
*TP53*	p53	Tumor suppressor	Li-Fraumeni syndrome	Breast, lung, and briain cancer; sarcomas; leukemia
*PTEN*	PTEN	Tumor suppressor	Cowden syndrome	Hamartomatous polyps, lipomas, & ganglioneuromas. Benign and malignant thyroid & breast lesions.
*CDH1*	E-cadherin	Epithelial intercellular adhesion	Hereditary diffuse gastric cancer syndrome	Breast, gastric, & colorectal cancer.
*STK11*	STK11	Tumor suppressor	Peutz-Jeghers syndrome	Mucocutaneous pigmentation; breast, colon, lung, thyroid, pancreas, gonadal, and bladder cancer.
MMR Genes	MSH2, MLH1, MSH6, and others	Mismatch repair	Lynch syndrome	Breast, colon, endometrial, ovarian, gastric cancer.
*PALB2*	PALB2	Homologous recombination	N/A	Breast, ovarian, prostate, & pancreatic cancer.
*CHEK2*	CHK2	Tumor suppressor	N/A	Fanconi anemia; breast, thyroid, colon, & ovarian cancer.
*ATM*	ATM	Tumor suppressor	N/A	Ataxia-telengiectasia; breast & pancreatic cancer.

### What is the incidence of *BRCA* gene mutations? Are there populations with increased incidence?

The incidence of *BRCA* gene mutations in the general population is 0.2–0.3%.^12^ Three percent of women with breast cancer, 6% of women with breast cancer before the age of 40 years, and 10% of women with ovarian cancer are carriers of a *BRCA* mutation. Prevalence of *BRCA* mutations is higher among Ashkenazi Jewish women than non-Ashkenazi Jewish women, about 2%.^12^ Other populations with a higher prevalence of *BRCA* mutations include African Americans, as well as people from Iceland, Netherlands, Sweden, and some other European countries.^13^

### What is this patient's risk of having inherited the *BRCA1* mutation from her mother?

As *BRCA1* is a single autosomal dominant gene and the patient's mother is a known heterozygous carrier of a pathogenic nonsense variant, this patient's risk of inheriting the mutation from her mother is 50%. The patient's brother holds the same risk of inheriting the deleterious mutation.

### What are the current recommendations for testing for *BRCA* mutations?

Due in part to the decreasing cost of molecular testing, many physicians and professional groups recommend genetic testing be made available to all patients with a personal history of breast cancer or newly diagnosed breast cancer.^14^ However, the National Comprehensive Cancer Network (NCCN) recommends that only a high-risk subset of patients with diagnosed breast cancer undergo molecular testing for a germline pathogenic *BRCA* variant.^15^ Some of the factors that support germline testing include being diagnosed at a young age (≤45 years), with more than one breast cancer, or with triple-negative breast cancer. Additional characteristics supporting testing include having a personal history or close relatives with *BRCA*-related cancers or being from Ashkenazi Jewish ancestry. Once an individual is found to have a pathogenic/likely pathogenic *BRCA* variant, the NCCN recommends that any individual in their family be eligible for germline genetic testing, irrespective of the degree of relatedness.^15^

The NCCN also recommends that individuals who are personally unaffected by *BRCA*-related cancers but have a strong family history of these malignancies be eligible for testing.^15^ However, direct genetic testing of those family members affected by malignancy is preferred, if possible.

Deciding when to pursue molecular testing for *BRCA1/2* mutations or other cancer susceptibility genes can be difficult for physicians, even with the aid of one of the several available *BRCA* risk assessment tools. However, regardless of the clinical scenario and patient history, genetic counselors should be utilized to help decide if genetic testing is recommended, what type of testing should be done, and also to guide the patient and help them appropriately understand the results and utility of the test.

### How does *BRCA* mutational status correlate with breast histopathologic and molecular subtypes?

Historically, breast cancer treatment was based primarily on histopathologic features. More recently, however, molecular expression profiles guide treatment, and immunohistochemical (IHC) stains are used as surrogate markers for gene expression. Triple-negative breast cancer (TNBC) are tumors defined by the absence of estrogen receptor (ER) and progesterone receptor (PR) (only 1% of tumor cells must stain positive for either to be considered ER/PR positive), as well as lack of overexpression of HER2.^16^ These tumors tend to be poorly differentiated. Overall, TNBC accounts for 10–20% of invasive breast cancers, and most carcinomas in women with *BRCA1* mutations are triple negative. TNBC also tends to occur more frequently in African American patients and younger patients (<50).^17^

TNBC carries a poor prognosis; approximately 46% of TNBC patients will have a distant metastasis at the time of diagnosis and the 5-year mortality rate after diagnosis is 40%. Additionally, they have a predilection to relapse. However, while endocrine or immunologic therapy options may be limited in TNBC, the intrinsic inability of BRCA-deficient cells to undergo DNA repair makes breast cancer in BRCA patients particularly susceptible to poly (ADP-ribose) polymerase (PARP) inhibitors and platinum-based therapies such as cisplatin.^18^

## Diagnostic findings, Part 2

**Fig. 1:**
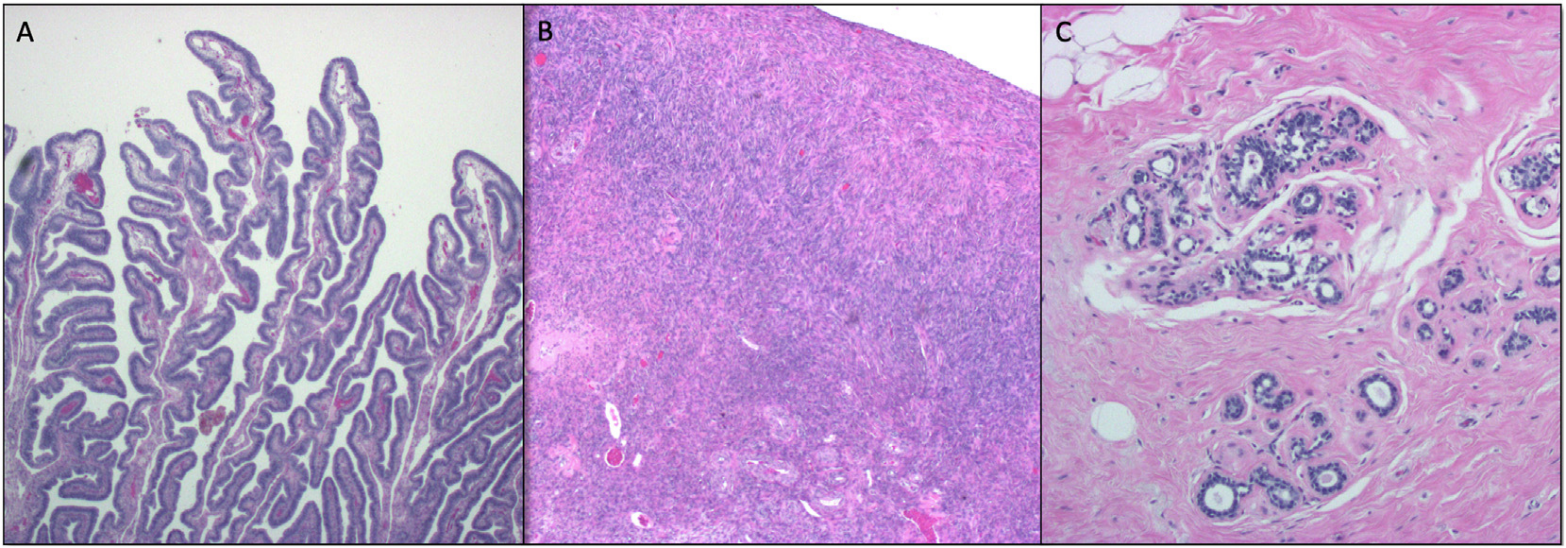
Representative histologic images of the patient's left fallopian tube (A, 2x), ovaries (B, 4x), and breasts (C, 10x). These tissue samples demonstrate normal histology.

**Fig. 2:**
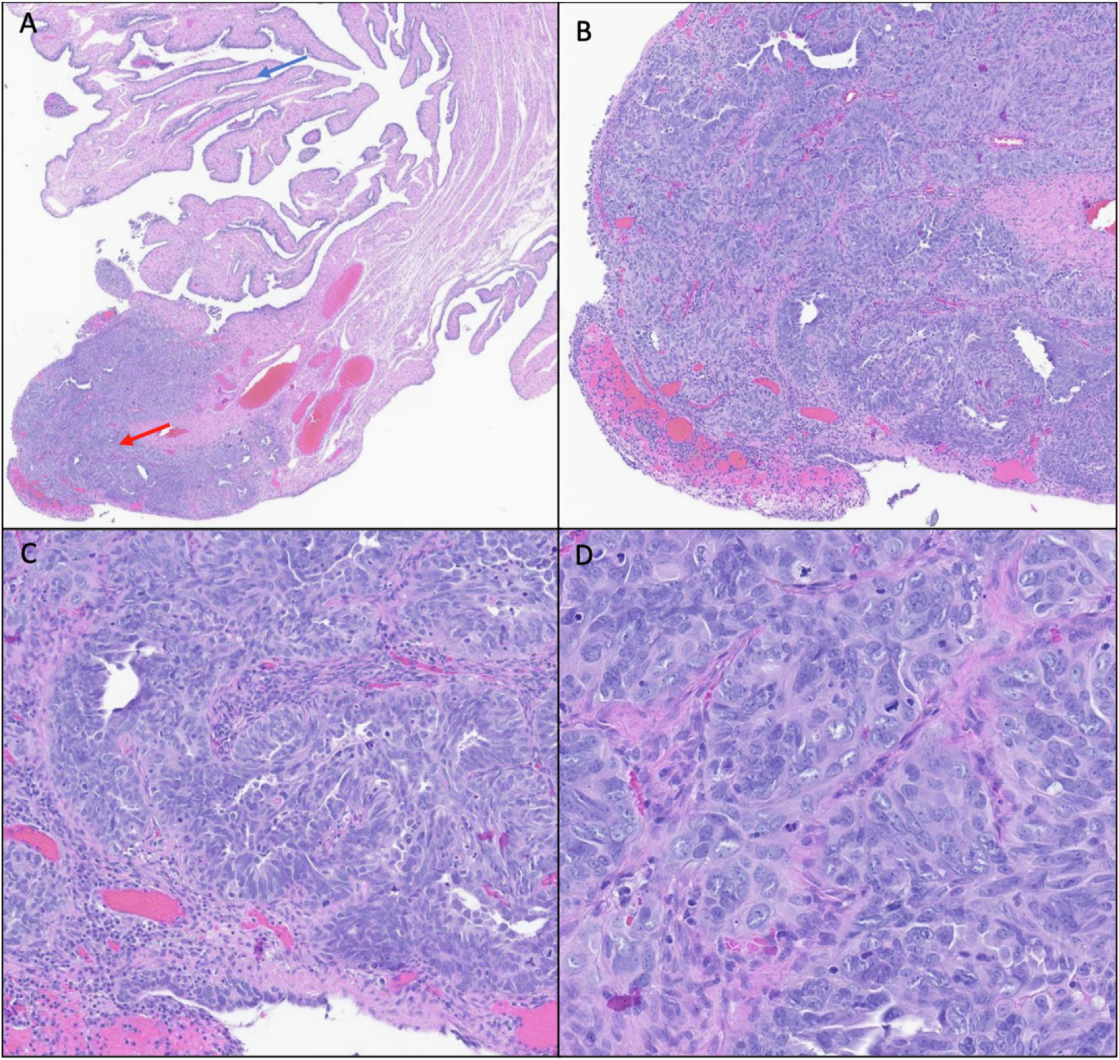
The fimbriated end of the fallopian tube shows sheets of pleomorphic mitotically active cells haphazardly arranged in crowded glands. Numerous mitoses can be appreciated. The cancerous cells appear confined to the tip of the fallopian tube (red arrow) (A, 2x; B, 5x; C, 10x; D, 20x). Blue arrow indicates area of normal histology.

The patient undergoes genetic testing, and the results show that she is a heterozygous carrier for the *BRCA1* mutation. These results are explained to the patient by the genetic counselor. The patient decides to undergo prophylactic bilateral mastectomy and salpingo-oophorectomy. The breast specimens, ovaries, and left fallopian tube are unremarkable (Fig. 1). Histologic images of the right fallopian tube are seen in Fig. 2A–D.

## Questions/discussion points, Part 2

### What are the management options for *BRCA*-positive patients in terms of additional screening and prevention?

*BRCA*-positive patients are counseled regarding the following options: surveillance, bilateral mastectomies, bilateral salpingo-oophorectomy, and chemoprevention.

Surveillance consists of a vigilant screening regimen in order to detect potential new malignancies at an early, treatable stage. It is currently recommended by the American Cancer Society that *BRCA* patients be screened yearly with MRI starting at age 25 and yearly with a mammogram starting at age 30. Patients should alternate these screening modalities once they reach age 30, therefore undergoing one screening every six months.^19^

Prophylactic mastectomy and/or bilateral salpingo-oophorectomy, as illustrated in this case, are also an option for these patients. The NCCN currently recommends prophylactic bilateral salpingo-oophorectomy after completion of childbearing between ages 35 and 40 for *BRCA1* carriers and between ages 40 and 45 for *BRCA2* carriers. Approximately 65% of *BRCA1* mutation carriers will have a risk-reducing salpingo-oophorectomy prior to their natural menopause.^15^^,^^20^ It should be noted, however, that these operations are risk-reducing, and not entirely preventive of cancer. Pre-operative discussions between the doctor and patient should include the impact of these surgeries on body image along with options for reconstructive surgery, as well as the impact of prophylactic bilateral salpingo-oophorectomies on patient's ability to become pregnant, the effects of surgical menopause.

Chemoprevention by risk-reducing medications such as estrogen receptor modulators (i.e. tamoxifen or raloxifene) or aromatase inhibitors is another option. Oral contraceptives have also been shown to decrease the risk of ovarian cancer in *BRCA1* and *BRCA2* carriers.^21^ There are a number of additional preventive measures that a patient can take to reduce their risk of developing cancer. These measures include lifestyle changes, such as exercising regularly and maintaining a healthy diet. The extent of risk reduction of these measures has not been shown to be as significant as the other options described.

### What are the histopathologic findings of the fallopian tube and ovary? Explain how these findings are related to this patient's *BRCA1* mutation

At the fimbriated end of the fallopian tube, a dense population of pleomorphic, mitotically active cells haphazardly arranged in crowded glandular structures is identified; this is high-grade serous carcinoma (Fig. 2). However, the ovary shows no evidence of malignancy.

This finding of carcinoma in the fallopian tube with unaffected ovaries is not unexpected in this *BRCA1*-positive patient. This is because recent clinical and molecular evidence has shown that fallopian tube epithelium, *not* ovarian epithelium, is the origin of high-grade serous ovarian carcinoma, and spread to the ovary occurs via direct contact with the malignant fallopian tube epithelium.^16^ This occurs in a particularly high proportion of high-grade serous carcinomas that develop in *BRCA*-positive patients. Evidence for this paradigm shift began to accumulate after serous tubal intraepithelial carcinoma (STIC), a precursor to serous carcinoma, was discovered in a large subset of *BRCA* patients who had prophylactic salpingo-oophorectomy, most commonly in the fimbriated ends of the fallopian tube. Additional studies showed that STIC was commonly seen in patients diagnosed with serous ovarian carcinoma and that these lesions were genetically related.^22^ Given this new insight into the pathogenesis of tubo-ovarian cancer in *BRCA* carriers, some have proposed prophylactic salpingectomy with delayed oophorectomy be offered to these patients to maintain child-bearing abilities and delay menopause.^23^

Overall, high-grade serous carcinoma is the most common type of cancer that affects the ovary, accounting for nearly 70% of all malignancies at this site.^18^ It is also the only type of tubo-ovarian cancer that BRCA carriers are at an increased risk of developing. Of all patients diagnosed with high-grade serous carcinoma, 15–20% have a germline *BRCA* mutation.^24^

Fortunately for this patient, the fallopian tube carcinoma appears to have been removed before spreading to other sites, and 5-year survival rates approach 100% for stage I fallopian tube serous carcinoma and over 90% when confined to the ovary.^25^ At more advanced stages, the prognosis for high-grade serous carcinoma is poor; 5-year survival for those with metastatic disease is only 25–30%.^22^

Upon further workup, it is confirmed that the carcinoma was contained to the removed fallopian tube. At her follow-up appointment six months later, she is doing well and reports that her mother's cancer has responded favorably to a PARP inhibitor and platinum-based chemotherapy regimen.

## Teaching points

• *BRCA1* and *BRCA2* are tumor suppressor genes whose products participate in double-stranded breaks in DNA via homologous recombination. Pathogenic germline mutations in these genes increase one's risk of breast, tubo-ovarian, prostate, pancreatic, and primary peritoneal cancers. While *BRCA* genes are the most well-known to increase the risk of breast cancer, germline mutations in many other genes can also increase the risk.
• There are a number of criteria that must be considered when discussing molecular testing for germline *BRCA* mutations. Genetic counselors should be utilized in these discussions. Overall, the incidence of *BRCA* mutations in the general population is 0.2–0.3%, with certain populations, most notably the Ashkenazi Jewish population, being at increased risk. *BRCA* patients comprise 3% of all breast cancer diagnoses in women, as individuals with *BRCA1* or *BRCA2* mutations have a 40–65% risk of developing breast cancer by age 70.
• Recent evidence has shown that high-grade serous carcinoma associated with *BRCA* mutations is of fallopian tube origin, with ovarian involvement occurring later. Preventive and screening measures should be taken with known *BRCA* carriers, as prognosis worsens significantly with increased breast and tubo-ovarian tumor stage.

## Funding

The article processing fee for this article was funded by an Open Access Award given by the Society of ‘67, which supports the mission of the 10.13039/100016205Association of Pathology Chairs to produce the next generation of outstanding investigators and educational scholars in the field of pathology. This award helps to promote the publication of high-quality original scholarship in *Academic Pathology* by authors at an early stage of academic development.

## Declaration of competing interest

The authors declare the following financial interests/personal relationships which may be considered as potential competing interests: Jerome P. Givi reports financial support was provided by Society of ’67.
